# Precise regulation of RAS-Mediated PI3Kα activation: therapeutic potential of BBO-10203 in cancer treatment

**DOI:** 10.1186/s40164-025-00706-8

**Published:** 2025-09-29

**Authors:** Ziyi Fan, Erqing Tan, Bin Song

**Affiliations:** Third Hospital of Shanxi Medical University, Shanxi Bethune Hospital, Shanxi Academy of Medical Sciences, Tongji Shanxi Hospital, Taiyuan, 030032 China

**Keywords:** RAS-PI3Kα signaling pathway, BBO-10203, Protein-Protein interaction inhibitor

## Abstract

In recent years, the Phosphoinositide-3-Kinase α (PI3Kα) signaling pathway has been increasingly recognized as a critical driver of tumorigenesis, particularly in breast cancer drug resistance and other solid tumors. Although conventional PI3Kα inhibitors (e.g., Alpelisib) have shown efficacy in extending progression-free survival in patients with PI3Kα-mutant breast cancer, their clinical application remains constrained by off-target toxicities, particularly hyperglycemia, which limits dosing and therapeutic feasibility. Building on recent preclinical findings, this study introduces BBO-10203, a first-in-class, orally bioavailable small-molecule inhibitor targeting the RAS-PI3Kα interaction. The compound is rationally designed to selectively and covalently bind to Cysteine 242 (Cys242) within the Rat Sarcoma (RAS)-Binding Domain (RBD) of PI3Kα, thereby effectively disrupting RAS-mediated PI3Kα activation. This unique mechanism confers potent in vivo antitumor activity while preserving insulin-regulated glucose metabolism, thereby mitigating metabolic adverse effects.

RAS protein cycles between active and inactive states through binding to Guanosine Triphosphate (GTP) or Guanosine Diphosphate (GDP), respectively. In its GTP-bound state (RAS-ON), RAS facilitates activation of downstream signaling pathways. Within the RAS-PI3K-AKT-mTOR signaling axis, RAS binds to and activates PI3K, triggering the generation of Phosphatidylinositol 3,4,5-Trisphosphate (PIP3), which subsequently activates Serine-Threonine Protein Kinase (AKT) and mammalian Target of Rapamycin (mTOR), thereby regulating essential cellular processes including growth, metabolism, and anti-apoptotic mechanisms [1, 2]. The PI3Kα signaling pathway functions as a central driver of tumor initiation, progression, and therapeutic resistance. Dysregulation of PI3Kα signaling not only promotes uncontrolled cell growth, proliferation, and metabolic reprogramming but also contributes to resistance in solid tumors, including breast cancer—particularly Estrogen Receptor-Positive, Human Epidermal Growth Factor Receptor 2-amplified, and PI3Kα-mutant subtypes—against conventional therapies [3–6]. Although therapeutic strategies targeting direct PI3Kα kinase inhibition have shown clinical efficacy, dose-limiting metabolic toxicities, including hyperglycemia and hyperinsulinemia, substantially constrain their broader application [3, 7]. Consequently, there is a critical need to develop novel therapeutic approaches that selectively suppress PI3Kα signaling in tumors while preserving insulin-dependent PI3Kα activation in normal tissues.

## Innovative mechanism of BBO-10203

**Fig. 1:**
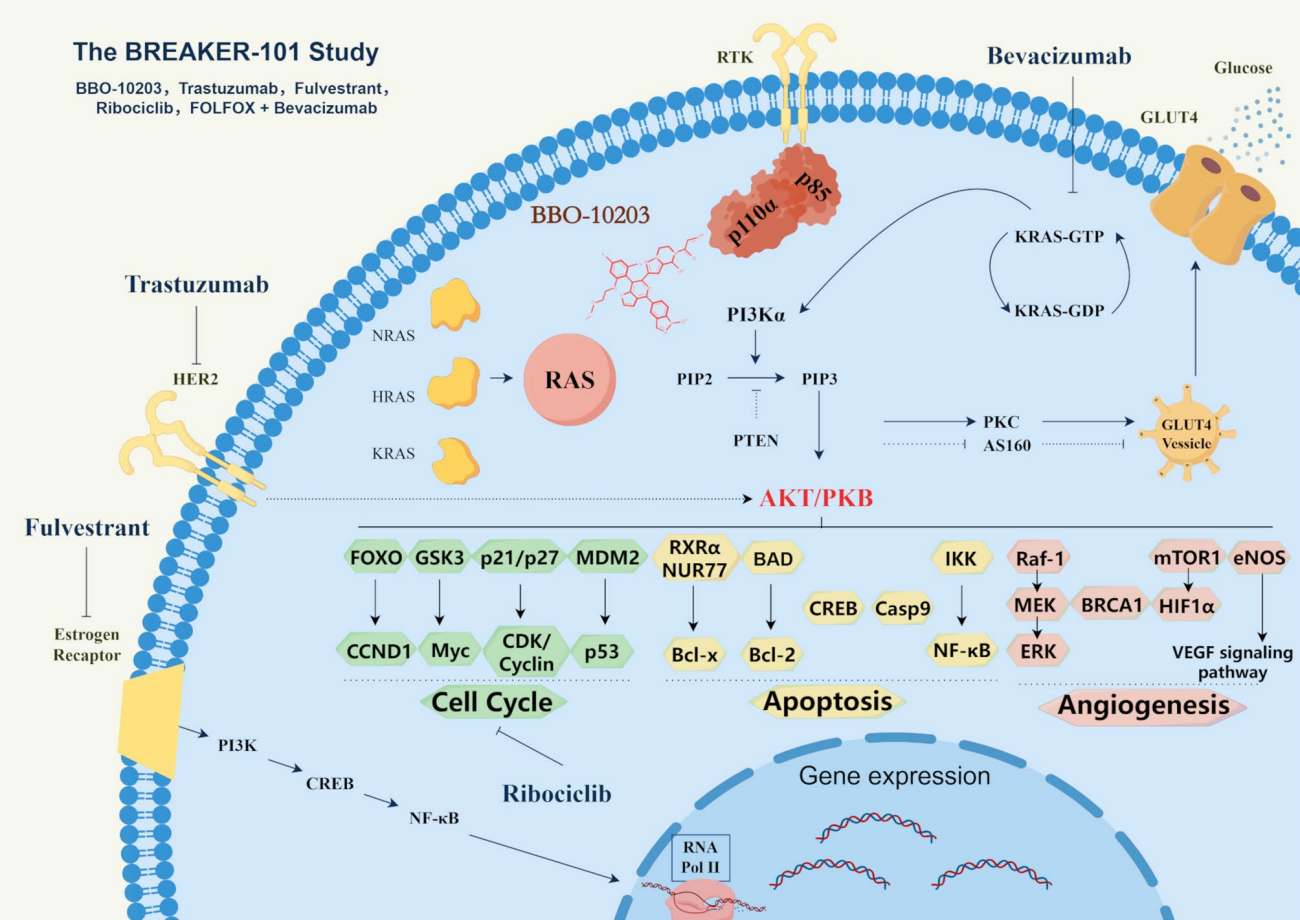
The PI3K-AKT axis regulates cell cycle progression, apoptosis, angiogenesis, and glucose transport, with glucose uptake mediated by PI3Kα activation independently of RAS. BBO-10203 selectively and covalently binds to the RBD of the p110α subunit, thereby specifically blocking RAS-dependent pathway activation without affecting glucose metabolism. The diagram illustrates the combination regimens currently under clinical trials involving BBO-10203, including Trastuzumab (anti-HER2 monoclonal antibody), Fulvestrant (estrogen receptor antagonist), Ribociclib (CDK4/6 inhibitor), and Bevacizumab (anti-VEGF antibody) combined with FOLFOX chemotherapy in KRASmutant tumors. (Created by Figdraw). Abbreviations: FOLFOX = folinic acid, fluorouracil & oxaliplatin; GLUT4 = Glucose Transporter Type 4; AS160 = AKT Substrate of 160 kDa; NUR77 = Nuclear receptor NR4A1; RXRα = Retinoid X Receptor alpha; eNOS = Endothelial nitric oxide synthase; RNA Pol II = RNA Polymerase II

As a first-in-class, orally bioavailable covalent small molecule, BBO-10203 represents a paradigm shift in antitumor drug development, transitioning from conventional kinase activity inhibition to precise upstream modulation of signaling pathways. In contrast to conventional PI3Kα kinase inhibitors that directly engage the kinase active site, BBO-10203 selectively and covalently binds the critical Cys242 residue within the RBD of the PI3Kα catalytic subunit p110α, thereby specifically disrupting the interaction between RAS isoforms (K-RAS, H-RAS, and N-RAS) and PI3Kα [3, 4, 7, 8]. This innovative strategy not only enhances the precision of antitumor signaling inhibition but also substantially reduces the risk of perturbing glucose metabolism in normal cells, thereby broadening the potential therapeutic window (Fig. 1).

BBO-10203 demonstrates potent inhibition of phospho-AKT (pAKT) across multiple cell lines, with particularly high efficacy observed in BT-474 cells. In vivo, oral administration of BBO-10203 resulted in 80–88% tumor growth inhibition in KYSE-410 and BT-474 xenograft models, with sustained effects observed for up to 24 h [3, 7]. Unlike conventional PI3Kα inhibitors, which induce hyperglycemia through disruption of insulin signaling [9–11], BBO-10203 preserves glucose homeostasis without inducing hyperglycemia or hyperinsulinemia, as confirmed by oral glucose tolerance tests (OGTT) [3, 7]. With favorable pharmacokinetic characteristics and oral bioavailability, BBO-10203 is suitable for chronic dosing, and its dose- and time-dependent inhibition profiles inform clinical dose selection [3, 7].

## Combination therapy strategies and potential applications in drug-resistance mechanisms

Drug resistance continues to represent a major challenge in cancer therapy. Current evidence indicates that persistent activation of the AKT/mTOR pathway not only drives tumor cell proliferation but also underlies resistance to diverse anticancer therapies. Although single-target PI3K/AKT pathway inhibitors (e.g., Idelalisib, GSK2334470, and Ipatasertib) can rapidly suppress AKT activity within minutes, such inhibition is often transient, with AKT activity frequently rebounding, occasionally exceeding baseline levels within 24 hours. Simultaneous inhibition at multiple nodes of the pathway using a combination of three targeted inhibitors can, however, induce a more sustained suppression of AKT activity while reducing the effective dose required for each agent [12].

Within this context, BBO-10203 demonstrates synergistic potential in multiple combination therapies through effective blockade of RAS-mediated PI3Kα activation. In combination with the anti-HER2 antibody Trastuzumab, BBO-10203 exhibited pronounced synergistic antitumor effects in HER2-positive breast cancer models [7]. Similarly, in HER2-positive breast cancer models, co-administration with selective estrogen receptor degraders (SERDs, e.g., Fulvestrant) or CDK4/6 inhibitors (e.g., Palbociclib) markedly enhanced antitumor activity, in certain cases resulting in tumor stasis or regression [7]. Furthermore, BBO-10203 demonstrated promising therapeutic benefits in combination with novel RAS inhibitors (e.g., BBO-8520 and BBO-11818) in KRAS-mutant lung cancer and other tumor models [13]. This multi-targeted combination strategy offers valuable insights into overcoming single-agent resistance and provides a theoretical basis for the development of personalized precision therapies [1, 3, 4, 7, 8, 13]. Currently, BBO-10203 has advanced to Phase 1 clinical trials (NCT06625775, Table 1).

**Table 1:** A Phase 1a/1b Open-Label Study Evaluating the Safety, Tolerability, Pharmacokinetics, and Efficacy of BBO-10203 in Subjects With Advanced Solid Tumors (The BREAKER-101 Study)

Participant Group	Details	Drug Intervention	Drug Administration	Cancer type
Experimental: BBO-10203	Participants enrolled in this cohort will receive BBO-10203 tablets orally (different dose levels will be evaluated) once daily as monotherapy. This cohort will enroll patients with HER2-positive advanced breast cancer, HR-positive HER2-negative advanced breast cancer, advanced colorectal cancer, and advanced lung cancer.	Drug: BBO-10203	Participants will receive assigned dose of BBO-10203 orally once daily	Breast Cancer, Colorectal Cancer and Lung Cancer
Experimental: BBO-10203 + Trastuzumab	Participants enrolled in this cohort will receive BBO-10203 tablets orally in combination with trastuzumab (8mg/kg infusion over 90 minutes on Cycle 1 Day 1, 6mg/kg infusion over 30–90 minutes during subsequent cycles or 600mg subcutaneous). This cohort will enroll patients with HER2-positive advanced breast cancer.	Drug: Trastuzumab	Participants will receive trastuzumab as infusion or subcutaneous injection every 21 days	Breast Cancer
		Drug: BBO-10203	Participants will receive assigned dose of BBO-10203 orally once daily	
Experimental: BBO-10203 + Fulvestrant	Participants enrolled in this cohort will receive BBO-10203 tablets orally in combination with fulvestrant (500mg IM). This cohort will enroll patients with HR-positive, HER2-negative advanced breast cancer.	Drug: BBO-10203	Participants will receive assigned dose of BBO-10203 orally once daily	Breast Cancer
		Drug: Fulvestrant	Patients will receive Fulvestrant as an intramuscular injection every 28 days (additional dose on C1D15)	
		Drug: Ribociclib	Patients will receive Ribociclib orally once a day (21 days on treatment, 7 days off)	
Experimental: BBO10203 + Fulvestrant + Ribociclib	Participants enrolled in this cohort will receive BBO-10203 tablets orally in combination with fulvestrant (500mg IM) and ribociclib (600mg orally) as determined in the dose escalation. This cohort will enroll patients with HR-positive, HER2-negative advanced breast cancer.	Drug: BBO-10203	Participants will receive assigned dose of BBO-10203 orally once daily	Breast Cancer
		Drug: Fulvestrant	Patients will receive Fulvestrant as an intramuscular injection every 28 days (additional dose on C1D15)	
		Drug: Ribociclib	Patients will receive Ribociclib orally once a day (21 days on treatment, 7 days off)	
Experimental: BBO10203 + FOLFOX + Bevacizumab	Participants enrolled in this cohort will receive BBO-10203 tablets orally in combination with FOLFOX (oxaliplatin 85 mg/m2, leucovorin 400 mg/m2, 5FU 400 mg/m2 + 2400 mg/m2) and bevacizumab (5 mg/kg IV). This cohort will enroll patients with KRAS-mutant advanced colorectal cancer.	Drug: FOLFOX	Patients will receive FOLFOX as infusion every 14 days	Colorectal Cancer
		Drug: Bevacizumab	Patients will receive bevacizumab as infusion every 28 days	
		Drug: BBO-10203	Participants will receive assigned dose of BBO-10203 orally once daily	

Recently, structural biology has assumed an increasingly pivotal role in elucidating protein-protein interactions [14]. Structural insights into the RAS-p110α complex, as reported by Daniel J. Czyzyk et al., were instrumental in guiding the rational design of BBO-10203 [4]. These findings not only provided a theoretical foundation for the successful development of BBO-10203 but also established a strategic framework for the design of future small-molecule inhibitors targeting protein-protein interactions.

## Conclusion

As a small-molecule inhibitor targeting protein–protein interactions (PPI), BBO-10203 has demonstrated potent antitumor activity in preclinical models, with the potential to provide safer and more effective therapeutic options for PI3K-driven tumors. Preclinical findings indicate that BBO-10203 may represent a first-in-class PI3Kα-targeted agent, conferring dual advantages of robust antitumor efficacy and reduced metabolic toxicity through selective inhibition of the RAS-PI3Kα interaction. This approach may offer novel therapeutic opportunities for patients with RAS-mutant or PI3K active solid tumors. Although research is currently confined to the preclinical stage, BBO-10203 has exhibited substantial promise with respect to efficacy, pharmacokinetics/pharmacodynamics, and safety. Future investigations should prioritize clinical development, elucidation of resistance mechanisms, exploration of rational combination strategies, and expansion of its therapeutic applications. Moreover, studies addressing its potential in combination regimens, strategies to overcome acquired resistance, and precision applications-particularly in ER+/PIK3CA-mutant and HER2-amplified malignancies-will be of critical importance. Achieving safe and effective therapeutic targeting of the RAS and PI3K pathways continues to represent a major challenge. Detection of the RAS-PI3Kα complex may facilitate validation of the target dependency of BBO-10203, while monitoring dynamic changes in key downstream effectors, such as AKT phosphorylation, could provide pharmacodynamic readouts for efficacy assessment and potential biomarkers to guide personalized precision therapy.

## Data Availability

No datasets were generated or analysed during the current study.

## Abbreviations

PI3Kα: Phosphoinositide 3-Kinasesα
Cys242: Cysteine 242
RBD: RAS-Binding Domain
RAS: Rat Sarcoma
GTP: Guanosine Triphosphate
GDP: Guanosine Diphosphate
PIP3: Phosphatidylinositol 3,4,5-Trisphosphates
KRAS: Kirsten Rat Sarcoma
AKT: Serine-Threonine Protein Kinase
pAKT: Phospho-AKT
ER: Estrogen Receptor Positive
HER2: Human Epidermal Growth Factor Receptor 2
OGTT: Oral Glucose Tolerance Test
PPI: Protein-Protein Interaction
